# Does domiciliary welfare rights advice improve health-related quality of life in independent-living, socio-economically disadvantaged people aged ≥60 years? Randomised controlled trial, economic and process evaluations in the North East of England

**DOI:** 10.1371/journal.pone.0209560

**Published:** 2019-01-10

**Authors:** Denise Howel, Suzanne Moffatt, Catherine Haighton, Andrew Bryant, Frauke Becker, Melanie Steer, Sarah Lawson, Terry Aspray, Eugene M. G. Milne, Luke Vale, Elaine McColl, Martin White

**Affiliations:** 1 Institute of Health & Society, Newcastle University, Newcastle upon Tyne, United Kingdom; 2 Department of Social Work, Education & Community Wellbeing, Northumbria University, Newcastle upon Tyne, United Kingdom; 3 Health Economics Research Centre, University of Oxford, Oxford, United Kingdom; 4 Institute of Cellular Medicine, Newcastle University, Newcastle upon Tyne, United Kingdom; 5 Newcastle City Council, Newcastle upon Tyne, United Kingdom; 6 MRC Epidemiology Unit, University of Cambridge, Cambridge, United Kingdom; TNO, NETHERLANDS

## Abstract

**Background:**

There are major socio-economic gradients in health that could be influenced by increasing personal resources. Welfare rights advice can enhance resources but has not been rigorously evaluated for health-related impacts.

**Methods:**

Randomised, wait-list controlled trial with individual allocation, stratified by general practice, of welfare rights advice and assistance with benefit entitlements, delivered in participants’ homes by trained advisors. Control was usual care. Participants were volunteers sampled from among all those aged ≥60 years registered with general practices in socio-economically deprived areas of north east England. Outcomes at 24 months were: CASP-19 score (primary), a measure of health-related quality of life; changes in income, social and physical function, and cost-effectiveness (secondary). Intention to treat analysis compared outcomes using multiple regression, with adjustment for stratification and key covariates. Qualitative interviews with purposive samples from both trial arms were thematically analysed.

**Findings:**

Of 3912 individuals approached, 755 consented and were randomised (381 Intervention, 374 Control). Results refer to outcomes at 24 months, with data available on 562 (74.4%) participants. Intervention was received as intended by 335 (88%), with 84 (22%) awarded additional benefit entitlements; 46 did not receive any welfare rights advice, and none of these were awarded additional benefits. Mean CASP-19 scores were 42.9 (Intervention) and 42.4 (Control) (adjusted mean difference 0.3 [95%CI -0.8, 1.5]). There were no significant differences in secondary outcomes except Intervention participants reported receiving more care at home at 24m (53.7 (Intervention) vs 42.0 (Control) hours/week (adjusted mean difference 26.3 [95%CIs 0.8, 56.1]). Exploratory analyses did not support an intervention effect and economic evaluation suggested the intervention was unlikely to be cost-effective. Qualitative data from 50 interviews suggested there were improvements in quality of life among those receiving additional benefits.

**Conclusions:**

We found no effects on health outcomes; fewer participants than anticipated received additional benefit entitlements, and participants were more affluent than expected. Our findings do not support delivery of domiciliary welfare rights advice to achieve the health outcomes assessed in this population. However, better intervention targeting may reveal worthwhile health impacts.

## Background

Socio-economic inequalities in health are universally observed and persist into old age.[1–3] Older people, especially those in poor health, may need additional income or support, such as payments for social care, transport, domestic help and aids and adaptations to their home to maintain their health and independence.[4] Such social welfare benefits are hypothesised to improve health-related quality of life, mediated by reduced stress, the adoption of more advantageous social arrangements and healthier behaviours. These changes in turn are hypothesised to enable greater choice and control over life circumstances, leading to healthier choices and greater independence.[4–7] Historically in the UK, there has been substantial under-claiming of such financial and non-financial social welfare benefits among those with low incomes and poor health.[8, 9] While observational epidemiological evidence on the socio-economic patterning of health is strong, rigorous studies evaluating the impact of increasing financial or material resources on health outcomes are rare.[5] Ecological studies examining the reunification of Germany suggest important impacts of increased income on mortality, although other factors may have been influential.[10–12] A systematic review of 10 North American randomised controlled trials (RCTs) of income supplementation experiments targeting a range of age groups, carried out in the late 1960s and 1970s, showed that none had reliably assessed the effects of increased income on health.[13] Although such experiments are unlikely to be repeated, one way of assessing the health impact of increasing financial resources would be to evaluate the impact of assisting claimants to obtain previously unclaimed welfare entitlements.[13] The RCT reported here aimed to evaluate an intervention designed to maximise welfare benefit uptake among independent living older people in order to test the hypothesis that access to additional resources might improve health outcomes.

Around the world, countries support those with additional needs through a range of measures, including income subsidies, material benefits and services, which may be available to all, be means tested or only accessible according to other criteria, such as age or health status. In the UK, there is a wide range of welfare benefits available for older people, but uptake of entitlements is sub-optimal.[8, 9] For example, under-claiming of Pension Credit is reported to be around 33% and under-claiming of Council Tax Benefit around 40%.[14] Entitlement to one benefit often acts as a ‘passport’ to others, and many of the benefits aimed at older people are linked together. This presents a complex network of entitlements that for potential claimants can be difficult to navigate and access without expert assistance.[4–7] In the UK, services have been developed to provide such assistance, including those offered by local government social services departments and voluntary organisations, such as Citizens’ Advice and Age UK. Eligibility for health-related benefits (and failure to claim) increases with age, particularly post retirement, so the study reported here focused on people aged 60 and over.[6, 15, 16]

We conducted a systematic review to assess the impact of such services on benefit uptake and health outcomes.[5] The review identified numerous studies that demonstrated the financial and material benefits of such welfare rights advice (WRA) services. Increased access to welfare entitlements was associated with ‘active assistance’ with benefit claims, in particular for older people who may struggle with complex forms and bureaucracy. Many advice services were offered in a healthcare context (mostly primary health care). However, only two studies evaluated health outcomes.[17, 18] Neither study employed a rigorous experimental design and they were not therefore able to determine with any degree of certainty whether access to additional resources had a positive impact on health. Nevertheless, these studies provide insights into the potential benefits of such interventions and further qualitative studies have identified a range of potential positive outcomes in physical, behavioural and psycho-social domains of health.[4–6, 19]

This study was preceded by a pilot RCT. [19–21] The intervention was developed in close collaboration with a local government social services department that wished to reduce under-claiming of benefits among the older population in relatively socio-economically deprived areas. In this pilot RCT, 58% of participants were awarded either financial (median gain £55/week (US$82, €82), non-financial (e.g. aids and adaptations to the home) or both types of benefits,[19] confirming the feasibility and success of the intervention from the point of view of accessing unclaimed benefits. We identified a number of key design and methodological issues, which we address in this evaluation. These are discussed in detail in elsewhere. [22] However, of particular note was the considerable time that may elapse between the first advice session and the receipt of new financial or material benefits, particularly in complex cases or if there is an appeal. Since any health benefits may well not be seen immediately, we extended the follow-up period from 12 months in the pilot RCT to 24 months in this full scale evaluation.

## Methods

### Research governance

We report the study according to the Consolidated Standards of Reporting Trials (CONSORT) statement.[23] The study protocol was prospectively registered (Current Controlled Trials ISRCTN Number: 37380518) and published.[22]

All fieldwork complied with the UK National Research Ethics Service,[24] Caldicott guidelines[25] and the Data Protection Act 1998.[26] This study was reviewed and approved by the UK National Research Ethics Service Committee, South West–Exeter (reference number 11/SW/0260).

### Study design

We conducted a pragmatic, individually randomised, single-blinded (researchers), parallel-group, wait-list controlled trial of domiciliary WRA versus usual care, with embedded economic, and quantitative and qualitative process evaluations.

### Setting

The trial took place in eight of the 12 local government districts in socioeconomically disadvantaged areas in the North East of England. Social services departments (or their contractors) in these districts provided the domiciliary WRA intervention for the trial. We aimed to recruit two general practices (family doctor services) per district, sampled from among all general practices in each district, ranked according to a score for socio-economic deprivation for a defined geographic area using the 2010 English Index of Multiple Deprivation (IMD) calculated at middle-layer super output area (MSOA) level (an administrative geographical unit approximately equivalent in size to a parliamentary ward) for practice premises (main location) postcodes, in accordance with the method of Griffin et al.[27] Those general practices in the lower two-fifths of the deprivation ranking distribution, without existing dedicated or targeted WRA services, were eligible for inclusion. We sampled participants from these practices.

### Participants and recruitment

Participants were volunteers aged ≥60 years (one individual per household) who were not resident in a nursing home or in hospital, were not terminally ill (as assessed by their GP) and were fluent in written and spoken English. Participants were sampled randomly from among all those identified as eligible by their general practice and then invited by letter from the practice. The names and contact details of those not opting out at this stage were passed to the research team. Research interviewers contacted these individuals by telephone to arrange a face-to-face meeting at a mutually convenient time in the participant’s own home. Interviewers sought written informed consent and then proceeded to collect baseline data.

### Randomisation and blinding

Following baseline assessment, participants were randomised to either intervention or control condition, stratified by general practice. Sequential allocation tables for each practice were independently generated from random numbers prior to recruitment (by a statistician using Stata version 12 software [StataCorp, College Station, TX, USA]). Participants were allocated in the chronological sequence in which they were recruited and immediately sent a standard letter informing them of their group allocation. Only the project administrator had access to the allocation tables, and the allocation was concealed from the research team, data collectors and statisticians. The administrator immediately informed the appropriate local welfare rights advisor of the contact details of each newly allocated intervention group participant and requested that they should be visited for a welfare assessment within two weeks. Twenty-four months later, WRAs were sent lists of control group participants to assess once follow-up had been completed.

### Intervention

We report the intervention according to the Template for Intervention Description and Replication (TIDieR) guidelines (See S1 Text).[28] We summarise the intervention here and further details are presented in Table A in S1 Table. The intervention comprised face-to-face WRA consultations and active assistance with benefit claims, delivered in participants’ own homes and tailored to their individual needs by a qualified welfare rights advisor employed by local government departments, or their contracted services. In one local government district, WRA services were contracted and delivered by a voluntary organisation. Participants underwent a full benefit entitlement assessment involving assessment of financial, material and welfare status; assessment of previous benefit entitlement and claims; discussion of current entitlement and options for action, including new claims (financial and non-financial). Active assistance with benefit claims and other welfare issues was given, which included completion of benefit application forms on behalf of participants. Participants were followed up at home or by telephone as required until they no longer required assistance. We optimised the intervention prior to delivery by providing information, training and guidance for welfare rights advisors and GPs on their respective roles in ensuring welfare entitlements.

### Intervention receipt

Intervention receipt was assessed as the proportion of those eligible to receive the intervention who actually received it. The causes of participants not receiving the intervention as intended and reasons given by participants for this were recorded. We used IMD 2010 scores, assigned at household level by matching postcode to IMD score at lower super-output area (LSOA) level,[29] to examine the socio-economic patterning of receipt of the intervention and of welfare benefits. Rates of eligibility and receipt of welfare benefits, broken down by type of benefit, were expressed as a proportion of those assessed by welfare rights advisors in the intervention group at baseline. The rates were also assessed in the control group at the 24-month follow-up.

### Quality control and fidelity assessment

Intervention procedure checklists were given to all welfare rights advisors to ensure consistent delivery. We asked welfare rights advisors to record the date and time of each initial WRA assessment, to assess whether or not these were delivered within 2 weeks of baseline data collection. We further assessed whether or not initial WRA assessments were delivered as intended by analysing audio-recordings of welfare rights advisors undertaking intervention delivery against a checklist in a subsample of participants, selected for convenience by welfare rights advisors. We aimed to analyse one initial welfare advice consultation per welfare rights advisor (n = 19). Audio-recorded consultations were assessed by a senior welfare rights advisor from a local government department not involved in the trial.

### Comparator (wait-list control condition)

Participants randomised to the control group received ‘usual care’ (standard practice) from both health and WRA services after randomisation until they had completed their 24-month follow-up assessment. They were given no advice regarding welfare rights as a part of the study intervention during this period, but were free to seek WRA independently from a local government or voluntary sector provider at any time. Participants who sought independent advice remained in the trial and were analysed in the control arm on the intention-to-treat principle, and details of any advice and ensuing claims and outcomes were recorded at 24-months. Following the 24-month assessment, participants in the control arm received the intervention, as delivered to the intervention group, including all visits to participants by welfare rights advisors and assistance with claims and appeals until all claims had been resolved.

### Data collection

Data were collected by interview in participants’ own homes at baseline and 24 months follow-up and by postal questionnaire (CASP-19 only) at 12 months. The primary outcome measure was health-related quality of life, measured using the CASP-19 scale (range 0–57),[30, 31] which is assessed using 19 questions in four domains: Control, Autonomy, Self-realisation and Pleasure. Reasons for the choice of this instrument have been described in detail elsewhere.[22]

Details of secondary outcomes are given in Table B in S1 Table. Social and demographic variables, including age, sex, ethnicity, educational level attained, employment status and living arrangements (number of household members, paying for accommodation, and whether or not emotional support was available), were collected to characterise the trial participants and adjust for potential confounding in analyses. We also measured functional ability, using the modified Townsend Activities of Daily Living scale,[32] and a life events score, calculated by recording eight potentially serious events, including bereavement and significant illness, that might have occurred in the past 7 months, as well as their impact on the individual.[33]

### Sample size

Power calculations showed a minimum of 318 participants needed to provide data at follow-up in each study arm to provide 90% power at 5% significance level to detect a 1.5-unit difference in mean CASP-19 score at 24 months between the intervention and control groups, assuming a standard deviation (SD) of 8.7 and a correlation between baseline and 24 months of 0.74.[30, 31, 34] Estimates of SD and correlation coefficients were derived from the results of English Longitudinal Survey of Ageing (ELSA) (wave 4), restricting the analyses to those aged ≥ 60 years[35], which also suggested a 1.5 unit difference would be clinically significant.[22, 34] Assuming an attrition rate between baseline and 24-month follow-up of 15% (as experienced in our pilot RCT),[19] we needed to recruit 750 participants to the study (375 to each group).

### Trial data analysis

Analyses used the intention-to-treat population, which comprised all participants in the group to which they were randomised, regardless of which intervention they received.

As interviewers collected most of the data, it was expected that there would be minimal missing data on items in scales. Unless specified otherwise by the scale developers, when no more than 20% of items were missing or uninterpretable on specific scales, scores were calculated by using the mean value of the respondent-specific completed responses on the rest of the scale to replace the missing items (simple imputation).[36]

For the primary outcome (CASP-19), multiple imputation, using iterative chained equations, was used to obtain a complete data set for the primary outcome at 12 and 24 months, conditional on survival to 12 or 24 months.[37] The variables considered for the multiple imputation model were those thought *a priori* to be associated with CASP-19 at 12 and 24 months, as well as variables that were predictors of missingness of CASP-19 scores.[38] The final model included baseline characteristics (age, gender, education, living alone), and CASP-19 score at baseline and 12 months. Twenty multiple imputation data sets were produced.

The primary analysis of all study end points was undertaken after applying the simple imputation method described above. The results of the CASP-19 are also reported after using multiple imputation.

Baseline characteristics of the study population were summarised separately within each randomised group, including primary and secondary outcome variables and covariates. No significance testing for any baseline imbalance was carried out, but any noted differences are reported descriptively.

When covariates were available in both the trial data set and the national ELSA survey (wave 4),[35] their distributions were compared to investigate the representativeness of the trial participants. The numbers and percentages of any financial and non-financial benefits (e.g. aids and adaptions) received since baseline were summarised separately within each of the randomised groups.

The primary end point, CASP-19, was compared at 24 months between the intervention and control groups using multiple linear regression with adjustment for baseline value and general practice (the stratification variable). The results are reported as a difference in means with a 95% confidence interval (CI). An adjusted analysis included the life events score and functional ability score at 24 months, and baseline covariates age, gender, education and marital status in the regression model.

Bootstrap estimation was used if the distribution was skewed. A similar comparison was also made at 12 months (in this analysis, the life events and functional ability scores at baseline were used instead of those at 24 months).

Each secondary outcome was compared at 24 months between the intervention and control groups using multiple linear regression with adjustment for baseline and general practice. The results are reported as a difference in means with a 95% CI. The adjusted analyses also included the baseline covariates of age, gender, education and marital status in the regression model. Bootstrap estimation was used for CIs when the distribution was skewed.

We used logistic regression with adjustment for general practice to compare proportions between the intervention and control group for categorical outcome variables. The results are reported as odds ratios with 95% CIs. An adjusted analysis in the regression model also included general practice, age, gender, education and marital status.

Exploratory analyses were performed in which the linear model for the primary outcome contained terms for intervention, other key variables (sex, age in years and education) and the interaction between them. In addition, within the intervention group, multiple linear regression explored whether the mean primary outcome (CASP-19) at 24 months differed, first between those receiving and not receiving WRA, and then between those receiving and not receiving any extra welfare benefits. A comparison was also made between the CASP-19 scores at 24 months for those in the intervention arm who had previously been awarded a financial welfare benefit and those in the control arm who were later awarded a financial benefit. As they were all eligible for financial welfare benefits, these participants should have been similar in their socio-economic and health profiles. All of these models also included baseline CASP-19 score, general practice, age, gender, level of educational attainment and marital status.

All statistical analyses were undertaken using Stata version 12 software [StataCorp, College Station, TX, USA]).

### Economic evaluation

The relative efficiency of the intervention was assessed by within-trial cost-consequences and cost-utility analyses.[39] Cost consequences analysis is increasingly used in evaluation of complex public health interventions that have multiple possible effects. It involves a descriptive presentation of the range of costs and benefits of an intervention, allowing the reader to form their own opinion on relevance and relative importance of the findings to their decision making context.[40]

The cost-utility analysis combined cost data and health-related quality of life, measured using the Euroqol (EQ-5D-3L) instrument[41, 42] at baseline and 24 months follow-up to estimate the incremental cost per quality-adjusted life-year (QALY) gained. Estimates of cost took the perspectives of the public sector services (for the service delivery costs of the trial intervention) and the UK Treasury (for additional benefits awarded). Data on the costs of the intervention were derived from records of activities and time dedicated to tasks kept by welfare rights advisors (casework contact sheets (CCSs)), standard salary scales, standard travel reimbursement rates, and resources used. Sensitivity analyses were performed to assess the impact of different data sources and varying key assumptions and parameters used in the economic evaluation. Given the likely skewed distribution of the cost data, bootstrapping was used to produce 95% CIs.

The duration of financial benefits paid to participants in the intervention group during the trial period was calculated as the number of weeks between the date of the award as documented by welfare rights advisors and the end of the study follow-up. As data on the duration of each financial benefit were not collected for the control group, we inferred durations for financial benefits received by participants in the control group using the median durations in the intervention group.

Unlike the financial benefits, for which weekly amounts per benefit were reported by participants, unit costs were not collected during the trial for the non-financial benefits. Participating local authorities and public sources provided information on unit costs for aids and adaptations that were part of the non-financial benefits and listed separately in the baseline and 24-month questionnaires. The Department of Health’s Community Equipment Services National Catalogue and Prescription Scheme[43] was used to provide nationally representative unit cost data for Community Equipment Services, which were used to calculate cost implications in absence of unit cost data from the participating local authorities.

As the period of study was two years, all outcomes (financial, non-financial, health-related quality of life) occurring in the second year were discounted at 1.5%, the recommended rate for public health interventions in the UK.[44] Changes in health-related quality of life over the 24-month trial period were captured by means of the EQ-5D-3L questionnaire.[41] Using the UK population tariff,[45] responses to the EQ-5D-3L questionnaire were converted into scores of participant-specific health state utilities at each time point. The EQ-5D-3L scores were then transformed into QALYs using the ‘area under the curve’ method.[45] From this, the mean QALY score for each group was calculated, along with incremental QALYs gained to capture the change in health-related quality of life between arms over the trial period. The difference in mean QALYs gained between the two trial arms were estimated both without adjusting for baseline characteristics and with adjusting QALYs for baseline EQ-5D-3L, age, and gender, to account for any imbalance in the characteristics between the two groups at baseline. Imprecision surrounding incremental QALYs was estimated using bootstrapping and the level of imprecision was presented as 95% CIs.[40]

The economic analyses estimated differences in costs and outcomes between the intervention and usual care (control group). All data were analysed in Stata version 13 software [StataCorp, College Station, TX, USA]).

The cost-utility analysis combined cost data and health-related quality of life measures to estimate the incremental cost per QALY gained by the intervention group compared with the control group at 24 months. The incremental cost per QALY for the intervention, compared with usual care, was estimated using seemingly unrelated regression (SUR), controlling for age, gender and baseline EQ-5D-3L.[46] This allowed for the simultaneous estimation of costs and QALYs gained, which were calculated at individual level, and accounted for unobserved individual characteristics that could affect both costs and QALYs and lead to the potential correlation of these two variables. Using SUR controlled for the potential bias in estimates and ensured efficient estimation.[47] Results were based on 1000 bootstrap iterations. For each iteration, the SUR analysis was run on the data set, non-parametric bootstrap samples were drawn from the SUR residuals for both trial arms; predicted values of incremental costs and QALYs were calculated using the bootstrapped residuals, and differences in mean costs and QALYs between the intervention and control groups were estimated with 95% CIs to account for uncertainty surrounding the incremental cost-effectiveness ratio (ICER). The ICERs calculated were compared against willingness-to-pay thresholds of relevance to UK decision-makers [i.e. the National Institute for Health and Care Excellence (NICE)’s current threshold of £20,000–30,000 as society’s willingness to pay (WTP) for one QALY gained].[48]

For the cost-consequences analysis, costs and various consequences of the intervention were assessed separately.[39] The implications for participants’ income, based on additional benefits received during the 24-month trial period as well as changes in EQ-5D-3L, were disaggregated and presented in the form of a balance sheet. In the balance sheet, outcomes are reported depending on which arm of the trial they favoured. Quantitative findings are presented as mean difference (for financial benefits, aids and adaptations, QALYs) or difference in the number of non-financial benefits between trial arms. Qualitative findings are also included in the balance sheet, thus synthesizing results from all aspects of the study. Further details of the economic evaluation methods are available from the corresponding author.

### Qualitative study

Semi-structured interviews were conducted with 50 purposively sampled participants identified from the trial database and recruited to achieve a maximum variation sample with respect to group allocation, gender, age, receipt of benefits and any unanticipated consequences of the intervention identified at follow-up. Sampling and interviews continued until data saturation was achieved.[49] Interviews were supported by a topic guide (see S2 Text) and explored the perceived impacts of the intervention, beneficial or otherwise. All interviews were digitally audio-recorded (with permission) and transcribed verbatim. Data were anonymised, and pseudonyms were applied. In this paper, we present findings from interviews with those who received additional benefits as a result of the intervention. Findings from other interviews will be reported elsewhere.

Data were analysed thematically using the Framework method[50] with constant comparison[51] and deviant case analysis[52] to enhance validity. NVivo 10 software (QSR International, Warrington, UK) was used to code and manage the data. Qualitative data were collected and analysed iteratively; themes that emerged in early interviews were explored in later ones. A coding framework was developed, applied to the first 10 interviews, and then revised taking into account initial insights. Three interviews were independently double-coded to ensure reliability of the coding framework, which was then applied to remaining interview data.

## Results

**Fig 1:**
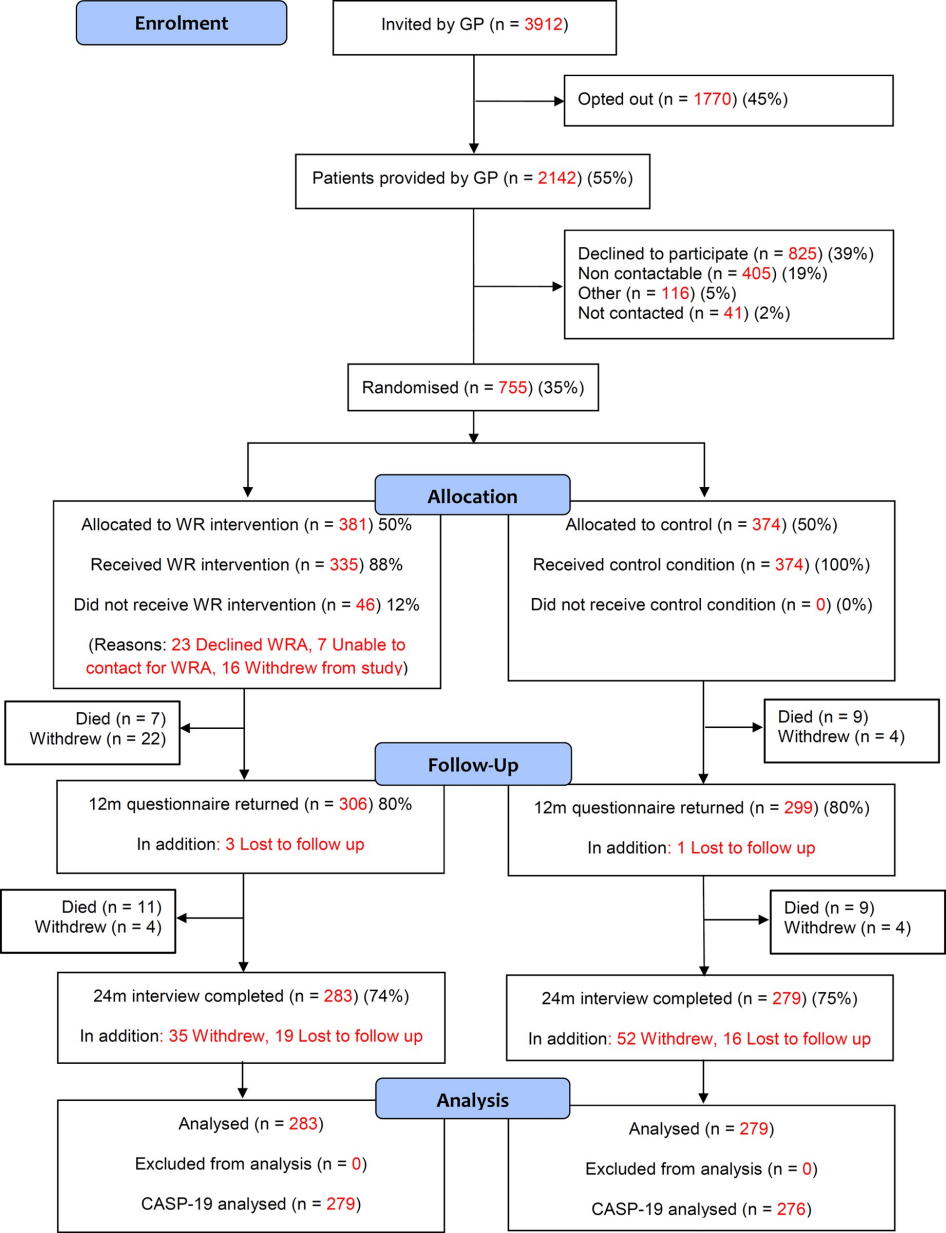
CONSORT [23] flow chart of participants in the RCT.

Three thousand nine hundred and twelve adults aged ≥ 60 years were approached by 17 general practices (Fig 1). Of these, 1770 (45%) opted out. The remaining 2142 (55% of those approached) expressed some interest in the study; their contact details were sent to the research team who invited them to participate. Of these, 825 (39%) declined to participate at this stage, 405 (19%) were not contactable and 41 (2%) were not contacted as the recruitment target had been reached before they were needed.

The remaining 755 agreed to participate, provided written consent, were assessed at baseline and were randomised to either intervention (n = 381) or control group (n = 374). Participants were, on average, less socio-economically disadvantaged (IMD score at LSOA level: 29.0 [SD 16.0]) compared with non-participants (n = 1387; IMD score: 33.5 [17.9]), and were more likely to be female (53.5%). Participants were recruited between 11/05/2012 and 28/02/2013.

During the trial, 121 participants withdrew from the study, 36 were lost to follow up and 36 participants died. Losses were balanced across allocation groups. In total, 562 participants completed 24 month follow-up (intervention group 283, control group 279) and were available for analysis.

### Receipt of intervention

The number of intervention arm participants seen as intended within 2 weeks by their allocated welfare rights advisor was 5 (1.5%) and within 4 weeks 37 (11%). The median number of days from study entry to first welfare rights advisor visit was 58 days (IQR: 40–89) and the range 0–403 days. The length of time taken for welfare rights advisors to see participants for their initial assessment increased as recruitment progressed. The median time from recruitment to welfare rights advisor case being closed was 83 days (IQR and range: 51–140.5 and 14–705 days, respectively).

Of the 381 intervention arm participants, 335 (88%) received the intervention as intended. For those not receiving the intervention (n = 46, 12%), the most commonly cited reason was that the participant declined the WRA consultation (n = 23); this represented 6% of those eligible and 50% of those not receiving the intervention. In addition, some participants withdrew from the study before intervention delivery (n = 16), while some could not be contacted in order to arrange the advice appointment (n = 7).

In total 84 of the 381 (22%) in the intervention arm were awarded an additional benefit, 25% of the 335 who received WRA. Forty six (12%) of the 381 intervention participants did not receive any WRA, and none of these were awarded welfare benefits (Table 1).

**Table 1:** Benefits received and welfare rights advice awarded to intervention arm participants (n = 381).

		Number (%) of intervention arm participants receiving new welfare benefits		
		**Yes**	**No**	**Total**
**Number (%) of intervention arm participants receiving welfare rights advice**	**Yes**	84 (22)	251 (66)	335 (88)
	**No**	0 (0)	46 (12)	46 (12)
	**Total**	84 (22)	297 (78)	381 (100)

Table 2 shows the distribution of the benefits received for those who took up the offer of WRA. Non-means tested benefits were most commonly received. There were small numbers in each of the categories of different combinations of financial and non-financial benefits received.

**Table 2:** Distribution of benefits received by 24 months among the 335 participants receiving welfare rights advice in the intervention arm.

	Number (%)
**Type of benefit:**	
None	250 (74.6)
Financial	65 (19.4)
Non-financial (Aids and adaptions)	14 (4.2)
Both financial and non-financial	5 (1.5)
Not known	1 (0.3)
Any type of benefit	84 (22)
**Of 84 participants who received benefits:**	
***Means or non-means tested benefits*:**	
Means tested	16 (19.0)
Non-means tested	49 (58.0)
Both means- and non-means tested	19 (23.0)
***Combination of type of benefit and means or non-means tested*:**	
Financial (means tested)	16 (19.0)
Financial (non-means tested)	34 (40.0)
Both financial (means and non-means tested)	15 (18.0)
Aids and adaptions (non-means tested)	14 (16.5)
Both financial & aids and adaptions (non-means tested)^a^	2 (2.5)
Both financial & aids and adaptions(means and non-means tested)	3 (3.5)

^a^ Non-means tested is not-applicable to non-financial benefits

### Fidelity assessment

Seven recordings of welfare rights advisor initial assessments with participants in the intervention group were made available for fidelity assessment. All consultations were carried out systematically, were consistent with the protocol for intervention delivery and included appropriate assessment of financial and health status, and all relevant applications for eligible means and non-means tested awards and benefits.

### Baseline characteristics of trial participants

Intervention and control arms were well balanced in relation to baseline variables (Table 3). Roughly half the participants were male, living alone and not paying for accommodation. They were predominantly white, educated to secondary school level, retired, were never or ex-smokers, and had a satisfactory level of emotional support. The average age was 70 years, while typical Townsend Activities of Daily Living scales were relatively high and stressful life events scores were relatively low.

**Table 3:** Baseline demographic data by trial arm.

**Categorical variables**	**Categories**		**Intervention** **(n = 381)**			**Control** **(n = 374)**		
			**Number (%)**			**Number (%)**		
**Sex**	**Male**		174 (46.0)			179 (48.0)		
**Ethnicity**	**White**		375 (98.5)			374 (100)		
**Education level**	**Primary**		4 (1.0)			2 (0.5)		
	**Secondary**		316 (83.0)			321 (86.0)		
	**Tertiary**		61 (16.0)			51 (13.5)		
**Living alone**	**Yes**		177 (46.5)			178 (47.5)		
	**No**		204 (53.5)			196 (52.5)		
**Employment status**	**Employed**		47 (12.2)			39 (10.5)		
	**Unemployed**		16 (4.2)			19 (5.0)		
	**Retired**		279 (73.2)			275 (73.5)		
	**Other**		38 (10.0)			39 (10.5)		
	**Missing**		1 (0.2)			10 (0.5)		
**Accommodation**	**Not paying**		226 (59.2)			192 (51.5)		
	**Paying**		145 (38)			174 (46.5)		
	**Other**		10 (2.5)			7 (2.0)		
	**Missing**		1 (0.25)			0 (0)		
**Need care at home**	**Yes**		97 (25.5)			115 (31.0)		
**Emotional support**	**Yes**		361 (94.8)			352 (94.0)		
	**No**		11 (2.75)			17 (4.5)		
	**Missing**		9 (2.5)			5 (1.5)		
**Smoking status**	**Never smoked**		124 (33.0)			118 (31.5)		
	**Ex occasional smoker**		30 (8.0)			29 (8.0)		
	**Ex daily smoker**		161 (42.0)			145 (39.0)		
	**Occasional smoker**		8 (2.0)			9 (2.0)		
	**Daily smoker**		58 (15.0)			73 (19.5)		
**Continuous variables (possible range)**	**n**	**Mean**	**SD**	**Observed Range**	**n**	**Mean**	**SD**	**Observed Range**
**Age in years**	381	70.6	7.1	60–92	374	70.6	7.5	60–94
**Townsend ADL (0–16)**^**a**^	381	10.9	4.8	0–16	373	10.7	5.0	0–16
**Life events score (0–32)**^**b**^	370	4.6	4.1	0–16	371	4.4	4.2	0–24
**IMD score**^**c**^	379	29.3	16.5	3.2–74.8	373	28.7	15.5	3.2–74.8

^**a**^ Townsend activities of daily living: Low scores indicate less favourable outcome and high scores better outcome on the scale.

^**b**^ High scores indicate less favourable outcome and low scores better outcome on the scale.

^c^ Index of Multiple deprivation: Higher scores indicate greater deprivation.

Table 4 shows the distribution of the primary and secondary outcomes at baseline. Participants typically had values towards the more favourable end of each scale for health-related quality of life, affordability index, standard of living, depression and healthy diet; and towards the less favourable end of the scale for social interaction and physical activity (PASE). The distributions were well balanced between intervention and control arms.

Those who dropped out of the trial before the 24 month assessment on average tended to be a little older, slightly more likely to be male and to have lower average CASP-19 scores at baseline, signifying poorer health-related quality of life.

**Table 4:** Baseline data–primary and secondary outcome measures by trial arm.

	Intervention		Control		
Scale (possible range)	n	Mean* (SD)	n	Mean* (SD)	Observed range
**CASP-19 QoL score (0–57)**^**a**^	354	41.4 (10.5)	351	40.7 (10.9)	11.6–57
**PHQ-9 depression score (0–27)**^**b**^	372	4.4 (5.3)	366	4.6 (5.2)	0–24.8
**Affordability index (4–20)**^**b**^	375	13.0 (2.8)	366	13.2 (2.7)	4–20
**Standard of living index (0–24)**^**a**^	381	18.7 (2.5)	374	18.6 (2.5)	7–24
**Social interaction score (0–27)**^**a**^	381	9.9 (4.6)	373	9.5 (4.3)	0–24
**PASE score(0–400+)**^**a**^	380	101.3 (67.5)	373	102.4 (72.3)	0–325.5
**Diet score (15–75)**^**a**^	380	46.9 (6.9)	373	46.8 (6.8)	23–66
**EQ-5D-3L score (-0.59–1)**^**a**^	374	0.589 (0.332)	363	0.583 (0.356)	-0.594–1
**Units of alcohol in last week–All participants**^**b**^	369	6.7 (11.2)	367	6.3 (10.2)	0–84
**Units of alcohol in last week–drinkers**^**b**^	267	9.2 (12.3)	243	9.4 (11.3)	0–84
**Receiving home care (hrs /wk)**^**†**^^**b**^	85	48.1 (56.1)	100	53.6 (57.5)	1–168

* Unadjusted mean and SD using simple imputation; Individual scale items for incomplete questionnaires with a response rate of at least 80% were imputed using the mean value of the respondent-specific completed responses

^†^ For those participants receiving care only

^**a**^ Low scores indicate less favourable outcome and high scores better outcome on the scale

^**b**^ High scores indicate less favourable outcome and low scores better outcome on the scale

### Outcome analysis

#### Intention to treat analysis of primary and secondary outcomes

Table 5 shows the distribution of CASP-19 score at baseline, 12 and 24 months by trial arm for complete cases and after application of multiple imputation. At 24 months the mean CASP-19 scores were 42.9 in the intervention group and 42.4 in the control group: an adjusted mean difference of 0.3 (95%CI -0.8 to 1.5). The differences in mean CASP-19 between trial arms remained very similar at all time-points when multiple imputation was used to deal with missing primary outcomes for some participants.

**Table 5:** Primary outcome: CASP-19 scores at baseline, 12 months and 24 months by trial arm using complete cases and multiple imputation (MI).

CASP-19 score (possible range: 0–57)^a^		Intervention		Control		Difference
Time point analysed	Observed range	n	Mean^b^ (SD)	n	Mean^b^ (SD)	Adjusted difference in means (I-C) (95%CI)^c^
**At baseline**	7–57	354	41.4 (10.5)	351	40.7 (10.9)	n/a
**At baseline (Using MI**^**d**^**)**	7–57	381	41.3 (10.5)	374	40.8 (10.7)	n/a
**At 12 months**	9–57	300	38.2 (10.0)	295	37.4 (10.6)	0.6 (-0.7 to 1.8)
**At 12 months (Using MI**^**d**^**)**	9–57	371	37.9 (10.1)	365	36.9 (10.6)	0.4 (-0.7 to 1.5)
**At 24 months**	6–57	279	42.9 (10.1)	276	42.4 (10.4)	0.3 (-0.8 to 1.5)
**At 24 months (Using MI**^**d**^**)**	6–57	320	42.7 (10.3)	317	42.6 (10.1)	0.4 (-0.8 to 1.5)

^**a**^ Low scores indicate less favourable outcome and high scores better outcome on the scale

^**b**^ Unadjusted mean and SD using simple imputation; Individual scale items for incomplete questionnaires with a response rate of at least 80% were imputed using the mean value of the respondent-specific completed responses

^**c**^ 95% confidence interval for adjusted mean difference in multiple linear regression. Models were adjusted for baseline covariates age, gender, education, marital status, general practice and CASP-19 score, as well as life events score and Townsend ADL scores at 24 months.

^**d**^ Multiple imputation (MI) using chained equations and predictive mean matching. Imputation model included baseline (BL) characteristics age, sex, education and living alone as well as CASP-19 score at BL. The model for CASP-19 score at 24 months was additionally adjusted for CASP-19 score at 12 months after imputation.

An exploration of whether the difference in means between trial arms varied significantly between subgroups of participants was carried out by testing for interactions between trial arm and sex, age-group (dichotomised at median age—68.6 years) and educational group (primary or secondary versus tertiary): all interaction terms were not statistically significant (p-values 0.94, 0.15 and 0.22, respectively) and, therefore, dropped from the regression model.

There was no evidence of a statistically significant difference at 24 months in means between trial arms for any of the secondary outcome scales or categorical variables (Table 6), other than for average hours of care received each week. The average hours/week of care were higher in the intervention arm (53.7 vs 42.0, adjusted difference of 26.3 hrs/week, 95%CI 0.8 to 56.1) However, for this variable, since few reported receiving care and the amount received varied from 1 to 168 hrs per week, estimates of the difference were imprecise and thus only of borderline statistical significance.

**Table 6:** Secondary outcome measures at 24m by trial arm.

**Continuous measures (possible range)**	**Intervention**		**Control**		**Difference**
	**N**	**Mean**^**a**^ **(SD)**	**N**	**Mean**^**a**^ **(SD)**	**Adjusted difference in means (I-C) (95% CI)**^**b**^
**PHQ-9 depression score (0–27)**^**d**^	278	3.9 (4.8)	276	3.9 (4.7)	0.2 (-0.4 to 0.9)
**Affordability index (4–20)**^**d**^	276	11.9 (2.3)	265	12.1 (2.2)	-0.1 (-0.5 to 0.3)
**Standard of living index (0–24)**^**c**^	283	18.9 (2.3)	279	18.7 (2.3)	0.1 (-0.2 to 0.3)
**Social interaction score (0–27)**^**c**^	282	10.5 (4.5)	277	10.3 (4.3)	0 (-0.5 to 0.5)
**Physical Activity Scale for the Elderly (PASE) (0–400+)**^**c**^	283	95.4 (59.8)	274	95.0 (60.6)	1.8 (-5.7 to 9.4)
**Diet score (15–75)**^**c**^	282	47.0 (6.2)	275	47.4 (6.2)	-0.2 (-1.0 to 0.6)
**EQ-5D-3L score (-0.59–1)**^**c**^	280	0.680 (0.296)	273	0.674 (0.318)	-0.015 (-0.057 to 0.028)^e^
**Units of alcohol in last week** **–ALL**^**d**^	281	6.3 (11.4)	278	6.1 (10.4)	0 (-1.3 to 1.2)
**Units of alcohol in last week–drinkers**^**d**^	200	8.8 (12.6)	183	9.2 (11.6)	-0.1 (-2.0 to 1.7)
**Receiving home care (hrs /wk)**^**d**^	42	53.7 (66.3)	52	42.0 (56.0)	26.3 (0.8 to 56.1)^e^
**Categorical measures (category)**	**Intervention (n = 283)**	**Control (n = 279)**	**Multivariable Logistic regression**^**f**^		
	**N**	**(%)**	**N**	**(%)**	**OR (95% CI)**
**Living independently (Dependent on others)**	52	(18)	56	(20)	0.9 (0.6 to 1.4)
**Mortality**^**g**^ **(Dead)**	18	(5)	18	(5)	1.11 (0.5 to 2.3)
**Smoking status (Increase since baseline)**	19	(7)	24	(9)	0.73 (0.4 to 1.4)
**See friends and relatives (Not as often as wished)**	63	(23)	65	(24)	0.9 (0.6 to 1.4)

^**a**^ Unadjusted mean and SD using simple imputation; Individual scale items for incomplete questionnaires with a response rate of at least 80% were imputed using the mean value of the respondent-specific completed responses

^**b**^ 95% confidence interval for adjusted mean difference in multiple linear regression. Models were adjusted for baseline score and general practice as well as baseline covariates age, gender, education and marital status.

^**c**^ Low scores indicate less favourable outcome and high scores better outcome on the scale

^**d**^ High scores indicate less favourable outcome and low scores better outcome on the scale

^e^ Distribution was positively skewed so bootstrap sampling was used to estimate 95% CI’s for adjusted difference in means

^f^ Logistic regression models adjusted for general practice (stratification variable) age, sex, education and marital status.

^g^ Mortality was based on full trial recruitment of 381 and 374 in intervention and control groups, respectively

### Exploratory analyses

A set of exploratory analyses looked at subgroups in the Intervention arm based on whether they received welfare advice or were awarded extra benefits. It would be expected that the socio-economic variables would not be balanced, as these factors are considered when an award is made. Table C in S1 Table shows the distribution of socio-demographic variables for those who did and did not receive any welfare benefits: there were no great differences, but those awarded welfare benefits tended to be slightly older, more likely to live alone and be female, and less likely to be well educated or able to carry out activities of daily living.

Table 7 shows that there was little difference in the crude mean total CASP-19 score at 24 months follow-up between those in the intervention group who did or did not receive WRA, with a small unadjusted difference in means favouring those not receiving WRA (42.8 for those receiving WRA vs 44.2 for those who did not). After adjustment for key covariates, the direction of difference did not change and was statistically non-significant (adjusted difference -2.1, 95%CI -5.5 to 1.3). The lower part of Table 7 shows that there was a lower average CASP-19 score at 24 month follow-up in those receiving benefits (mean total score 39.2 for those receiving benefits vs 43.8 for those who did not). This might be explained by the fact that those who received benefits tended to be worse off at baseline, in terms of health and socio-economic variables, than those who did not receive welfare benefits. After adjusting for these covariates, the difference in mean total CASP-19 score was smaller (adjusted difference: -0.7, 95%CI -2.8 to 1.4), and there was no indication of a statistically significant or clinically important improvement in average CASP-19 in those receiving welfare benefits.

**Table 7:** Comparing CASP-19 scores at 24 months between subgroups who did or did not receive welfare rights advice or welfare benefits (Intervention arm only).

	CASP-19				
	n	Mean (SD)^a^	n	Mean (SD)^a^	Adjusted difference in means (95% CI)^b^
***Welfare rights advice***	***Received***		***Not received***		***(Received–Not received)***
	261	42.8 (10.1)	18	44.2 (9.8)	-2.1 (-5.5 to 1.3)
***Welfare benefits***	***Awarded***		***Not awarded***		***(Awarded–Not awarded)***
	65	39.2 (9.4)	208	43.8 (10.1)	-0.7 (-2.8 to 1.4)

^a^ Unadjusted mean and SD using simple imputation; Individual scale items for incomplete questionnaires with a response rate of at least 80% were imputed using the mean value of the respondent-specific completed responses

^**b**^ 95% confidence interval for adjusted mean difference in multiple linear regression. Models were adjusted for CASP-19 baseline score and general practice as well as baseline covariates age, gender, education, marital status and life events and Townsend ADL scores at 24 months

Secondary outcomes were also compared between those who did or did not receive WRA or additional benefits. Table 8 shows that the adjusted differences in mean between the subgroups were all small and not statistically significant, except for the physical activity score (PASE). The adjusted mean PASE score was significantly higher in those who were not awarded any benefits (adjusted difference -13.9, 95%CI -27.0 to -0.9); this may reflect the better health of those who did not receive welfare benefits, rather than any effect of non-receipt of welfare benefits on the ability or choice to exercise more.

**Table 8:** Comparing secondary outcomes between subgroups of those who did or did not receive welfare rights advice or additional benefits (Intervention arm only).

Questionnaire score at 24 months possible range):	Welfare rights advice received	n	Mean (SD)^a^	Adjusted difference in means: received-not received (95% CI)^b^
**PHQ-9 depression score (0–27)**^**d**^	Yes	260	4.0 (4.7)	-0.6 (-2.5 to 1.2)
	No	18	3.0 (5.7)	
**Affordability index(4–20)**^**d**^	Yes	257	11.9 (3.0)	0.1 (-1.1 to 1.2)
	No	19	12.0 (3.4)	
**Standard of living index (0–24)**^**c**^	Yes	264	18.8 (2.3)	0.5 (-0.1 to 1.2)
	No	19	19.8 (2.5)	
**Social interaction score (0–27)**^**c**^	Yes	263	10.5 (4.5)	0.9 (-0.6 to 2.4)
	No	19	10.5 (4.6)	
**PASE score(0–400+)**^**c**^	Yes	264	94.6 (59.3)	0.2 (-22.1 to 22.6)
	No	19	106.4 (66.5)	
**Diet score (15–75)**^**c**^	Yes	264	47.0 (6.2)	-0.9 (-3.3 to 1.4)
	No	18	46.7 (6.9)	
**Units of alcohol in last week–All participants**^**d**^	Yes	262	6.3 (11.4)	1.0 (-3.1 to 5.0)
	No	19	5.7 (11.1)	
**Units of alcohol in last week–drinkers**^**d**^	Yes	187	8.9 (12.7)	1.0 (-5.4 to 7.4)
	No	13	8.3 (12.7)	
**Questionnaire score at 24 months possible range):**	**Benefits awarded**	**n**	**Mean (SD)a**	**Adjusted difference in means: awarded-not awarded (95% CI)**^**b**^
**PHQ-9 depression score (0–27)**^**d**^	Yes	65	5.2 (4.5)	0.6 (-0.5 to 1.7)
	No	207	3.5 (4.9)	
**Affordability index (4–20)**^**d**^	Yes	65	12.2 (2.1)	0.2 (-0.5 to 0.8)
	No	204	11.9 (2.3)	
**Standard of living index (0–24)**^**c**^	Yes	67	18.2 (2.1)	0.1 (-0.3 to 0.5)
	No	209	19.0 (2.4)	
**Social interaction score (0–27)**^**c**^	Yes	66	9.4 (4.2)	-0.1 (-1.0 to 0.8)
	No	209	10.8 (4.6)	
**PASE score(0–400+)**^**c**^	Yes	67	69.0 (45.3)	-13.9 (-27.0 to -0.9)
	No	209	103.0 (61.8)	
**Diet score (15–75)**^**c**^	Yes	67	46.4 (6.0)	-0.6 (-1.9 to 0.8)
	No	208	47.3 (6.3)	
**Units of alcohol in last week–All participants**^**d**^	Yes	67	5.9 (9.8)	0.1 (-2.4 to 2.5)
	No	207	6.3 (11.5)	
**Units of alcohol in last week–drinkers**^**d**^	Yes	44	9.0 (11.0)	0.7 (-3.0 to 4.4)
	No	149	8.7 (12.8)	

^a^ Unadjusted mean and SD using simple imputation; Individual scale items for incomplete questionnaires with a response rate of at least 80% were imputed using the mean value of the respondent-specific completed responses

^**b**^ 95% confidence interval for adjusted mean difference in multiple linear regression. Models were adjusted for baseline score and general practice as well as baseline covariates age, gender, education, marital status and life events and Townsend ADL scores at 24 months

^**c**^ Low scores indicate less favourable outcome and high scores better outcome on the scale

^**d**^ High scores indicate less favourable outcome and low scores better outcome on the scale

A further comparison was between the CASP-19 scores at 24 months for those in the intervention arm who had been awarded a financial welfare benefit and those in the control arm who were later awarded a financial benefit (after 24 month follow-up). Since they were all eligible for financial welfare benefits, these participants should be similar in their socio-economic and health profiles. The 55 participants in the intervention group who had been awarded financial benefits had a mean CASP-19 score of 39.2 (SD = 9.1), whereas the 48 in the control group (who were found to be eligible after the 24 month assessment) had a mean of 39.7 (SD = 9.4). After adjustment for covariates, the mean CASP-19 score was 1.4 higher in the control group (95%CI -2.0 to 4.7), suggesting there was no difference in CASP-19 between whose who had received benefits and those who did not.

We also looked at the relationship between CASP-19 and amount of welfare benefits received in the intervention group. It might be expected that those who had received greater benefits would have a higher CASP-19 score. The coefficient from simple linear regression model for CASP-19 on weekly amount received was -0.02 (95% CI: -0.08 to 0.04), showing a lack of association.

The final exploration looked at the association between the length of time for which a participant had been receiving additional benefits and CASP-19 score at 24 months. It might be expected that those who had received welfare benefits for a shorter period before the assessment would have shown less improvement in health, reflected in a smaller change in CASP-19 score. The vast majority (97%) of participants had been receiving benefits for at least a year by the time of final assessment and the correlation between CASP-19 score at 24 months and time from case being closed and the award of a benefit was 0.39 (95% CI: 0.16 to 0.58)–a weak positive association.

### Economic evaluation

The average total cost per participant for delivery of WRA was £44; 38% of these costs (£17) were travel costs associated with welfare rights advisors travelling to participants’ homes. The difference between intervention and control arm in the total mean amount for newly awarded financial benefits per participant was -£451 (95% CI: -£1,892; £991).

There was no evidence of statistically significant differences in the number of newly awarded non-financial benefits between trial arms, except that significantly more participants in the control group received general help with home insulation costs. There was no evidence of any significant differences in the number of newly awarded aids or adaptations between both trials arms with the exception of a ‘special telephone’ which was more frequently received in the intervention group.

The mean health gain was 0.009 (95% CI: -0.038, 0.055) QALYs and the incremental cost-effectiveness ratio (ICER) was £1,914/QALY gained. On average, the intervention was found to be more costly and more effective than usual care (Table 9). However, differences in QALYs gained between intervention and control groups were not significant (p = 0.966). The probability that the intervention would be cost-effective, should society be willing to pay £20,000 per QALY gained, was around 63%. This suggests that, given current evidence, in economic terms and taking a health service and local government perspective, we would be broadly indifferent to whether or not the intervention is implemented. From an individual perspective, however, the value of the additional benefits is a gain, albeit one which has not clearly translated into improvements in the measured, quantitative indicators of health over the 2 year time horizon of the study. These results were robust to changes in the discount rate and higher costs associated with the delivery of the intervention.

**Table 9:** Cost-utility analysis: Incremental cost-effectiveness ratio (ICER)—base case.

	Cost [£]	Incremental cost [£]	QALYs	Incremental QALYs	Incremental cost [£] per QALY gained (ICER)
**Control**	0.00		1.242		
**Intervention**	16.80	16.80	1.240	-0.002	Dominated
**Intervention adjusted (95% CI** ^**)**^^**a**^		17.18 (15.37, 19.05)		0.009 (-0.038, 0.055)	1,914

^a^ Results reported from SUR estimation; adjusting for baseline EQ-5D-3L, age and gender.

The results of the cost-consequence analysis are presented in a balance sheet (Table 10), indicating which arm of the intervention they favour (i.e. in which trial arm a significant positive difference in a particular benefit was observed). Table 10 also includes key qualitative findings, which are presented in greater detail in the next section (Qualitative findings). While there was no evidence of statistically significant differences between trial arms for most of the quantitative results, the qualitative findings suggested an improved quality of life among intervention group participants.

**Table 10:** Cost-consequence analysis–balance sheet.

**Outcome favours intervention (I)**	**Outcome favours control (C)**
*Qualitative findings* For some the nature of the intervention, involving a domiciliary visit and active assistance with claims, as well as reassurance concerning entitlement, relieved stress and generated positive feelings (*e*.*g*. peace of mind). For some the increased benefits allow the individual to escape a stressful and precarious financial situation. For some the increased benefits prevented the need for borrowing, reducing savings and helped reduce or prevent debt. Thus increasing financial security and reducing stress. For some the increased benefits alleviated food and fuel poverty and security against otherwise catastrophic unplanned costs. For some increased benefits helped to maintain mobility, independence and support formal and informal support with activities of daily living. For some increased benefits allowed the provision of monetary or non-monetary gifts for informal help received increasing perceptions of self-worth and reinforcing informal support networks.	*Financial benefits* *(mean difference in amount gained)*: Disability living allowance (mobility): £3,344 (95% CI: £2,654; £4,035) Carer’s allowance: £1,499 (£187; 2,810) *Non-financial benefits*: Insulation cost: An additional 16 participants received help with insulation cost (I: 24 (6%); C: 40 (11%); p = 0.030).
**No evidence that outcome differs between intervention and control group (I—C)**	
*No evidence of a difference in the following* *financial benefits* *(mean difference in amount gained) but confidence intervals wide enough to include economically important differences favouring either group*^*#*^: Average total amount: -£451 (-£1,892; £991) Council tax benefit: -£245 (-£694; £204) Housing benefit: -£250 (-£3,387; £2,886) Pension credit (guarantee): -£341 (-£2,653; £1,971) Pension credit (savings): -£83 (-£977; £811) Disability living allowance (care): £1,136 (-£2,742; £5,015) Attendance allowance (low rate): -£241 (-£684; £256) Attendance allowance (high rate): £174 (-£231; £580) Industrial injuries disablement benefit: -£494 (-£1,673; £685) *No evidence of a difference in the following* *non- financial benefits* *(difference in frequency between groups)*: *Non-financial benefits*^*#*^: Blue Badge: An additional 6 participants in the intervention group received Blue Badges (I: 21 (6%); C: 15 (4%); p = 0.313) Car: An additional 5 participants in the intervention group received a care from the Motability Scheme (I: 6 (2%); C: 1 (<1%); p = 0.057) Day centre attendance: An additional 4 participants in the control group attended a Day Centre (I: 2 (1%); C: 6 (2%); p = 0.150) Meals at home: The same number of participants received meals at home in both trial arms (I: 1 (<1%); C: 1 (<1%); p = 0.992) Grant from HEES: One additional participant in the intervention group received a grant from HEES (I: 10 (3%); C: 9 (2%); p = 0.828) Social tariff (electricity): One additional participant in the control group reported to be on a social tariff for electricity (I: 16 (4%); C: 17 (5%); p = 0.642) Financial help with optical prescription charges: An additional 5 participants in the control group received financial help with optical charges (I: 31 (8%); C: 36 (10%); p = 0.356) Financial help with dental treatment charges: An additional 6 participants in the control group received financial help with dental treatment charges (I: 17 (4%); C: 23 (6%); p = 0.266) *Aids and adaptations* *(mean difference in average total amount*, *95% CI)*^*#*^: Average total amount: £134 (-£582; £850) *Health-related quality of life* *(mean difference in QALYs gained*, *95% CI)*: QALYs gained: 0.009 (-0.038, 0.055)	

^#^ A positive difference implies that the average amount or the number of observations in the intervention group was greater than the average amount or number of observations in the control group.

### Qualitative findings

Interviews with trial participants suggested low levels of awareness of benefit entitlements among participants prior to the Intervention arm. Qualitative interview participants were sampled purposively, and so differed systematically from the overall sample. Mean CASP-19 score at 24 months follow-up for these qualitative participants was 38.7 (SD 7.9), which was a little lower than for the sample overall.

Of the fifty trial participants interviewed, 34 had made a successful financial benefit claim as a result of the intervention. A range of benefits was obtained (both means tested and health-related) and most participants were found to be entitled to more than one benefit. Two participants received non-means tested non-financial benefits including disabled parking badges, walking aids and home adaptations. Many participants talked about how the extra money had enabled them to escape a precarious financial situation. The extra income helped reduce debts as well as mitigate the use of finite savings. Some participants reported that extra income enabled them to start saving to afford future expenses, such as social care or funeral costs. (Box 1).

**Box 1. Impacts of additional benefits on participants’ lives.**

Harry, 71, Control, received £37 weekly Housing Benefit: [Regarding receiving extra benefit income] *It will make us [me] feel better because I’ll not be wondering ‘what do I have to cut out for to pay that bill*?*’*, *which I used to do … I’m not feeling the pinch as much now whereas before it was a bit of a struggle*.

Beatrice, 75, Intervention, received £139 weekly Attendance Allowance and Carer’s Allowance: *Them years you didn’t get help … so I just had to struggle on*, *I’m used to having no money*, *you know I had years and years just struggling through*, *coping*, *but now I’m getting this*, *it’s made a big*, *big difference to my life*.

Jim (with his partner, Caron), 68 Intervention, received £58 weekly Carers Allowance: *The savings … they just kept going down and down …*

Interviewer: *How well do you think you could manage without the extra income* [from the intervention] *now*?

Jim: *Well we just wouldn’t be able to manage as long*, *effectively*.

Audrey, 80, Intervention, received £139 weekly Attendance Allowance, Council Tax Benefit and Pension Credit: *I have more peace of mind about paying for the things that help whereas before I took it all out me savings money which obviously went down … so it has given me a lot of peace of mind in that sense*.

Tom, 72, Control, received £7 weekly Council Tax Benefit: *It’s a buffer zone that’s handy you know … It’s just left in the bank to accumulate for any eventuality*. *I put money aside for my funeral and my wife’s funeral*.

Elaine, 77, Intervention, received £139 weekly Attendance Allowance and Pension Credit: *Well I know it’s there if I need it*, *the extra … I know it’s there if I needed to fall back on it*, *you know*. *I mean they are talking about doing* [operating on] *this other knee*, *there might come a time when I might need somebody coming in a couple a times a day and have to pay them*, *so I know that that money is there to pay for them*.

Additional income increased ability to meet basic household bills, transport costs and domiciliary care services. Participants reported being more able to afford food and heating without having to worry about rising costs and better able to cope with larger expenses, such as replacing broken household items. Extra income was also used to pay for costs associated with travel, including taxis, trains, buses or a private car. Access to affordable transport was vital for engaging in daily activities, including attending hospital appointments, shopping, and socialising with friends and family. Many trial participants spent extra benefits income on formal or informal domiciliary help with cleaning, shopping or personal care in their homes. Some trial participants reported spending the money on home services to maintain, improve or adapt their properties. Affording services, household adaptations, as well as daily living aids, was important for those with ill health and disability and facilitated independent living. (Box 2).

**Box 2. Ways in which additional benefit income was spent**

Beatrice, 75, Intervention, received £139 weekly Attendance Allowance and Carers Allowance: *Well I can buy more food than I was able to do before … by the time you’ve paid for your heating and one thing and another it was a struggle to get food in*. *Sometimes I had to go and borrow something off the family … from their cupboards and that you know … but it has helped that way*.

Arthur, 69, Intervention, received £72 weekly Attendance Allowance and Pension Credit: *I’ve just had a freezer broke doon* [down], *a fridge broke doon* [down] *and the cooker broke … I would have probably had to get a loan out if I didn’t have these benefits to replace them*.

Maria, 80, Intervention, received £136 weekly Attendance Allowance, Pension Credit and a community care alarm: *Me sister had a stroke about a year ago and she’s in a home at* [place name]. *Well if one of the relatives can’t take me*, *then I get the taxi and its £14 there and back*. *I couldn’t do that before* [the intervention] *… she’s the only sister I got*, *she’s stuck in there*, *she can’t get out … and she just lived round the corner there you know so I’ve missed her*.

Audrey, 80, Intervention, received £138.93 Attendance Allowance, Council Tax Benefit and Pension Credit: *I pay a girl to come and just wait for me in the bathroom while I had me shower while I’m getting in and out just to see I’m all right and I just have to ring another girl*, *Chloe*, *she goes and does all me shopping for me ye know so I pay her*. *But that’s how I look at the Attendance Allowance is to help me with my quality of life*, *to improve it*.

Oliver, 82, Control, received £81 weekly Attendance Allowance: *It helped me a lot*. *I’ve gotten my fence fixed out the front and I’ve gotten this* [mobile thermostat] *to help me heating … plus I’ve gotten other radiators fitted … so it’s a lot warmer now … I’m nearly 83*, *plus me chest*, *this weather*, *it kills us*. *I’ve got that COPD*.

Stacy, 67, Control, received £50 weekly Pension Credit: [Due to receiving extra benefit income] *I bought myself a new microwave … because I can’t manage pots and pans … because I have got a lot of nerve damage in my left arm and hand … And*, *I did go out and buy myself a vacuum cleaner*, *one of these very lightweight ones because I can’t manage my old vac … it is brilliant*.

Sheila, 79, Control, received £91 weekly Attendance Allowance and Council Tax Benefit: *I'm going to have to re-do my bathroom*, *because I have difficulty getting out of the bath*. *In fact*, *three times I nearly failed … I'm back to where I started from*, *believe it or not*, *which is basin and flannel … the old-fashioned way before bathrooms were invented*.

Interviewer: *And is that something that you weren't able to do before* [receiving benefit income from the intervention]?

Sheila: *No*, *I didn't have the money to do that*.

The cumulative impact of being more able to afford household, transport and home care costs, as well as the receipt of home adaptations, walking aids and accessible parking badges had positive effects on participants’ ability to engage socially and consequently on their reported overall wellbeing and health. Participants reported improved mental wellbeing, better access to social support networks as well as increased ability to cope more independently with existing physical health problems. Participants reported how improved levels of income reduced stress, worry and anxiety that had been created by financial uncertainty. Many participants expressed feeling happier, or gaining ‘peace of mind’, after receiving extra benefit income. Decreased financial worries were also reported to have a positive impact on physical health. Narratives revealed trial participants’ desire to maximise activities and social opportunities with friendship and family support networks whilst still able. Increased independence to engage in social activities was reported by participants as having a positive impact on mental health. Participants had various long-term conditions, often multiple morbidities, and did not feel these physical illnesses would be remediated through financial or non-financial gains. However, they did report that the additional income enabled them to cope better with their health problems (Box 3).

**Box 3. Perceived impacts on well-being and health of additional welfare benefits.**

William, 83, Intervention, received £77 weekly Attendance Allowance, Council Tax Benefit and Pension Credit: *I used to worry at one time but now I don’t*, *I’ve got no worries at all now*. *I know I’ve got enough coming in each week to last me that week and put a little bit away for whatever*.

Stacy, 67, Control, received £50 weekly Pension Credit: *I think it had done wonders for me*, *it has taken off a lot of stress problems … Generally having that little bit of extra money does … improve health slightly because it takes the stress away*. *If you take the stress away you are not tensing up*, *you are not damaging any muscles*, *you are relaxing more and you are sleeping better*.

Interviewer: [Regarding receiving a walking aid from the intervention] *And does being able to go out and about and have freedom to move*, *do you think that links to your health*?

Diane (with Charles), 77, Intervention, received a wheeled Zimmer frame with seat): *It is*, *just getting out*, *I love getting out*, *I love talking to people*. *I go across the square and I see lots of people and he* [husband] *goes along the river and I think that keeps him*, *you know*. *He must have been away about 2 and a half hours this morning … I think just being*, *getting out and just chatting to people*. *I think because if you don’t*, *if you cut yourself off you just turn into a vegetable*.

Lydia, 71, Control, received £34 weekly Carers Allowance: [Receiving the extra benefit income] *It means I can have the heating on all day*, *without having to worry*, *because I know I’ve got that extra money to pay … I mean Clive* [husband] *just sits in a wheelchair all day*, *so he’s on the cold side and I keep thinking well I can't have this*. *I mean this heating has been on since eight o’clock this morning and it’s usually on till eleven o’clock at night you know*, *but I think well it doesn’t matter*, *now*, *as long as he’s comfortable*, *we’ve got the money to do it*, *so that’s fine … that is a big weight off my shoulders*.

## Discussion

### Summary of main findings

Only 22% of intervention arm participants received additional financial or non-financial benefits following domiciliary WRA: the remainder declined the advice, were awarded no benefits or declined those for which they were eligible. There was no significant difference in the CASP-19 score between intervention and control participants at 24 months, nor evidence that the difference varied among sub-groups distinguished by age, sex or socio-economic position.

None of our exploratory analyses suggested that there were important effects on measured outcomes as a result of the receipt of WRA in the intervention group. We did detect significantly higher levels of physical activity at 24 months among those who did not receive additional benefits compared with those who did. We also found a weak but positive correlation between CASP-19 score at 24 months and the amount of time since receipt of the benefit.

Economic evaluation indicated that, on average, the delivery of domiciliary WRA was found to be more costly and more effective than standard practice, with an incremental cost per QALY gained of £1,914. However, the probability that the intervention was cost-effective was only 63% when compared to conventional thresholds for society’s willingness to pay for a QALY gained (£20,000). In cost-consequences analysis, from an individual perspective, the value of the receipt of benefits was a gain, albeit one which did not clearly translate into improved health-related quality of life over the two year time horizon of the study. Imprecision around all estimates was high and analyses involving multiple imputation to account for missing data yielded differing conclusions, indicating the degree of uncertainty that existed.

Qualitative data suggested that receipt of additional financial and non-financial benefits was perceived as having a positive impact on health-related quality of life. Overall, the picture painted by the qualitative findings was one that suggested the intervention, when leading to additional financial or non-financial benefits, resulted in improvements in health-related quality of life with the potential to impact on physical or mental health, and which could lead to increased independence.

### Strengths and limitations of the study

This is the first randomised controlled trial to examine the impact of WRA on health outcomes[5] and the first to explore specifically their impact on older people when delivered in their own home.[22] We employed rigorous controls to ensure data quality, and blinding to minimise bias among data collectors. Primary and secondary outcomes were measured using validated scales, and were chosen on the basis of rigorous pilot work.[19, 20, 22] We demonstrated the potential of the CASP-19 scale to show a clinically important change over time in relevant groups in advance of the trial using analysis of national cohort study data.[34] The study was powered to demonstrate such a change as a result of the intervention. Intervention and control groups were balanced on all variables, indicating appropriate and effective randomisation.

The trial included a detailed process evaluation providing data to help to explain the trial and economic evaluation findings, which will be reported in further detail elsewhere. We assessed the fidelity of the intervention by recording and analysing a sample of welfare rights advisor interactions with clients. We were only able to record seven interactions. Whilst it is possible that welfare rights advisers who were not recorded may have delivered the service in a different way, resulting in systematically different outcomes, we think this is unlikely, as a key performance indicator for welfare rights advisors is income maximisation, and they are therefore highly motivated to identify client eligibility. The qualitative study was rigorously conducted, with systematic and independent double coding of data to enhance internal validity. The economic evaluation comprehensively explored the potential for cost-effectiveness employing both cost-utility and cost-consequences analyses. Sensitivity analyses assessed the impact of different data sources and varying key assumptions and parameters.

Forty-five percent of older people who were identified by general practices opted out after the initial invitation. We have no information on these 1770 potential participants, who may differ significantly from those who later participated (e.g. in terms of benefit eligibility or health status). Similarly, of the 2142 we invited to participate, only 35% agreed to be randomised. Compared to those who declined, those who participated were, on average, less likely to be socio-economically disadvantaged and more likely to be women. We compared these data with both the ELSA cohort and with equivalent data from our pilot study. Comparison with the pilot study data suggests that the trial participants were somewhat more affluent than expected, which is likely to have reduced their eligibility for benefit outcomes and may explain the lower than expected proportion gaining additional benefits.[19] However, comparison with the general population enrolled in the ELSA study showed that our participants were less affluent, being less likely to own their own homes, and be in employment.[35]

We lost 21% of participants through withdrawal or loss to follow-up, which was higher than anticipated from our pilot RCT.[19] Those who left the study had poorer health than those who remained at 24 months. We were unable to interview anyone who dropped out of the study, so the reasons for attrition remain unclear.

An important limitation was the lower than anticipated proportion of participants in the intervention arm (84/335, 22%) found to be eligible for additional benefits (and, similarly, after 24 month follow-up, in the control group). This had the consequence of significantly reducing the chance that we could detect an overall meaningful effect of the intervention, since any signal from the small number of those eligible was diluted in the intention to treat analyses. The relatively small numbers of new benefits recorded, and the variation in observed amounts, resulted in substantial imprecision around point estimates for the value of benefits.

We designed the study to avoid contamination between trial arms. Participants were individually randomised and those in the control group received no contact from a study welfare rights advisor until after 24 month follow-up. However, participants were free to seek WRA independently or to claim benefits independently during the course of the study. In data collected from the control group at 24 months, a larger than anticipated proportion reported new benefits that had not been reported at baseline. Where these were financial benefits, they must have either resulted from new benefit claims, new eligibility (e.g. determined by age) or be the result of misreporting. Where they were non-financial (e.g. aids and adaptations in the home), it is possible that they were acquired by means other than welfare claims. Our qualitative work did not shed further light on the source of these benefits, and the numbers and amounts of benefits reported, in particular non-financial, differed considerably between the 24 month interview and the forms completed by welfare rights advisors at 24 months. We are aware that during the time of the study, a range of other voluntary sector WRA service providers were operating in the North East. Family and friends can also provide important sources of advice on claiming benefits.

In our qualitative study, many participants referred to how benefits alleviated anxiety or worry. In our secondary outcomes measures we included the PHQ-9 Depression scale, but did not include a specific measure of anxiety, which is a limitation.

Finally, most likely as a result of the age range of the population and the trial period of two years, the number of participants lost to follow-up was relatively high, which was reflected, for example, by almost 23% of EQ-5D-3L data missing in each trial arm at 24 month follow-up. There was also evidence that those remaining in the study at 24 months were healthier than those who dropped out.

### Strengths and limitations in relation to previous studies

We designed the trial to overcome the main methodological weaknesses of previous research. These included: lack of randomisation or controls; a limited range of outcomes without clear theoretical justification; limited statistical power; short-term follow-up; lack of economic evaluation; and lack of process evaluation to help explain findings.[17, 18]^,^ [22]

We maximised the likelihood of successful claims among those eligible by: providing the intervention in people’s own homes, so as to avoid the necessity to travel for those with health problems;[6, 53] ensuring there was active assistance with claims, so as to avoid the significant challenges that people face in completing complicated claim forms;[4, 20, 54] and providing training, information and guidance for both welfare rights advisors and GPs in assisting claims, so as to ensure the WRA was delivered with maximal fidelity.[19, 20]

The intervention was targeted to ensure it was delivered efficiently to those likely to benefit most from the intervention. To achieve this, we identified general practices in the poorest areas of north east England, using methods from previous studies.[27] However, we found that this did not guarantee that either the general practice population or the individual participants were similarly socio-economically deprived (e.g. in the lower two fifths of the distribution of the Index of Multiple Deprivation (IMD)), since general practice premises may be located in an area not representative of their total catchment population. Alternative ways to target might include identifying individuals in the socio-economically poorest areas from all general practices to assess for benefit entitlement (although this may face similar recruitment challenges), or identifying patients with unclaimed benefit entitlements with defined problems referred to a range of health and social care services. The latter approach has been used successfully with cancer patients.[55]

Our qualitative findings were strikingly similar to those of our pilot RCT.[20] They demonstrated a range of potentially important impacts on health-related quality of life at an individual level, which we had anticipated might translate into measurable quantitative improvements in CASP-19 and secondary outcomes in this trial.

### Meaning of the study: Possible mechanisms and implications for policy and practice

Taken together, the findings of this study cannot provide sufficient evidence to support the commissioning of domiciliary WRA as a means to promote health among older people. Nevertheless, limitations of the study suggest that the intervention might have a potentially beneficial effect and that this might be cost-effective. The findings in relation to trial endpoints are surprising, given the qualitative findings which suggest important impacts on health-related quality of life at an individual level. There could be a range of explanations for this.

Our method of identifying suitable practices yielded ones that, although based in relatively poor areas (i.e. in the lower two fifths of IMD distribution), did not yield trial participants with equivalent levels of socio-economic deprivation. This may be because there were fewer such potential participants in practices than anticipated, or older people at the poorer end of the socio-economic spectrum were not contactable or were less willing to participate. It is also possible that, when compared with the time of our pilot trial, there were fewer older people entitled to unclaimed benefits. However, data collected nationally does not support this proposition.[14] It seems probable that our recruitment method failed to identify and engage those most likely to be in need of the intervention. This has important implications for future evaluations and for service delivery models. Research on more targeted approaches to identifying evaluation participants eligible for new benefits is warranted.

We identified a correlation, albeit relatively weak, between time since receipt of benefit and level of CASP-19 at 24 months in the intervention group. This coupled with the lack of overall effect at 24 months, and the longer than anticipated time (median 58 days–substantially greater than the intended 14 days) taken by welfare rights advisors to conduct initial assessments of participants in the intervention group, suggests that a follow up longer than 24 months may have made it possible to detect a stronger effect. Put simply, it is possible that a two year follow-up may be too short for health effects to become manifest after receipt of benefits.

The feasibility of such a long-term follow-up (e.g. 36–60 months) in a randomised trial may be problematic, since in qualitative work with older people during the design of this trial, participants felt that for control group participants to have to wait longer than 24 months for the intervention would not be acceptable.[22]

We chose outcome measures based on existing literature, as well as the findings of our prior qualitative research.[20, 21] This suggested that the receipt of additional benefits among those living in socio-economically disadvantaged circumstances might have its greatest impact on health-related quality of life. The CASP-19 measure[30, 31] captures four domains (Control, Autonomy, Self-realisation and Pleasure) that most closely mapped onto the theoretical constructs defined in our prior research.[20] Nevertheless, it is possible that CASP-19 failed to capture sufficiently strongly the domain(s) of health or health-related quality of life that are likely to result from the impacts of increased resources on health. Nevertheless, we also failed to detect any effect using measures of physical and mental health. However, our lack of an explicit outcome measure for anxiety is a limitation acknowledged above. The exact mechanisms of the relationship between access to additional resources and improved health remain unknown.

It is possible that the receipt of additional benefits failed to have any measureable effect on health or health-related quality of life. Although this may seem implausible, the context in which these impacts are expected to have occurred needs to be taken into account. Participants in this trial were aged ≥ 60 years, many of whom were already in poor health, suffering a range of long-term conditions, many with multi-morbidity. These may have resulted from a lifetime of exposure to unhealthy environments or behaviours, consequent upon social disadvantage. Their potential to improve, or to reduce the rate of decline, may therefore be severely constrained by their condition, such that the amounts of additional resources awarded were too little, and too late, to result in measureable impacts.

While it has proved all too easy to demonstrate strong socio-economic patterning of health by measures of socio-economic position in observational research,[2, 3] few studies have been able to show under experimental conditions that increasing access to resources results in better health.[13] While this may seem counterintuitive, it is important to remember that we do not yet have a clear understanding of the causal relationship between socio-economic factors and health outcomes. Some progress has been made in the last 20 or so years, with research identifying multiple potential pathways,[5] some of which may be interdependent, leading to multiple outcomes. Studies such as the trial reported here may have simply not measured the right combination of exposures, outcomes or confounding factors to be able to pick up a measureable signal.

Nevertheless, we did identify some differences between intervention and control arms at 24 months which offer tantalising signals that WRA may have an impact on health. The proportion benefitting from personal care in their home increased in the intervention group compared with controls, indicating that the intervention may have helped participants gain access to much needed care, which could help them maintain their independence and access to beneficial social relations. These findings were corroborated by the qualitative data, which provided ample evidence that the intervention led to valued outcomes among those who gained financial or non-financial benefits. There was some evidence that the longer participants had benefitted from additional financial or non-financial resources, the higher their CASP-19 score, an indication that impacts may have been developing over time. It is entirely plausible that the timescale for the development of measureable outcomes is longer than the 18–24 months allowed in this study from receipt of new benefits to measurement of outcomes. It is also important to note that the number in receipt of new benefits was considerably lower than anticipated from our pilot trial,[19] and thus the effect was substantially diluted in the intention-to-treat analysis.

If the intervention is effective, it is likely cost-effective. It proved remarkably cheap to deliver (£44/case), even in comparison with usual practice of welfare advice not delivered in people’s own homes (an average additional cost of £17 per person for welfare rights advisors’ travel time and distance). The estimated cost per QALY gained was £1914, well below the NICE threshold of £20,000.

Taking into account all of our findings, we remain equivocal about whether domiciliary WRA is effective as a health intervention as assessed in the context of this trial. Positive impacts may be identified with a longer follow up period. Important impacts may also be identified in further evaluations that overcome the shortcomings of this research. Nevertheless, for those in whom it yields unclaimed benefits, WRA remains an important social and economic intervention that should continue to be delivered by local government and third sector organisations. Given many of the unclaimed benefits for those over 60 years are health-related, health care providers should also continue opportunistically to identify and refer to social services patients they believe may be eligible for unclaimed benefits.

### Unanswered questions and future research

Whilst further fundamental research is needed to understand the causal pathways between socio-economic position and health, there is also an onus on the research community to evaluate the impacts of improved socio-economic circumstances on health outcomes. It would be valuable also to conduct further studies to explore the impact of worsening socio-economic circumstances on health, particularly in older people, since ecological study evidence suggests there may be important impacts of austerity measures that might be mitigated by interventions such as WRA.[56]

It is questionable whether a trial such as this could be replicated, recruiting a sample population in greater need (as assessed by eligibility) for welfare benefits. It would be important to identify participants on the basis of their own socio-economic position rather than (or perhaps in addition to) using an ecological measure as was used in this study. Recruitment and retention might be lower, and practical and analytical strategies would need to be adopted to minimise and mitigate the effects of the potential biases.

It seems unlikely on ethical grounds that a trial could be conducted with longer follow-up in order to see whether impacts of the intervention emerge over a period of years. However, an alternative form of evaluation could be conducted, perhaps taking advantage of a natural experiment[57] in which a cohort of older people, some of whom have and others have not claimed benefits to which they are entitled, are followed up over an extended period. Such a study could make use of routine data or an existing cohort study to assess outcomes and could explore the impact of differing periods in receipt of benefits on outcomes. However, without targeted WRA providing a means for those currently not claiming their entitlements to gain access to them, such a study might suffer from similar problems, since sufficient claimants will be needed to measure an effect.

Researchers would need to consider carefully the outcome measures of interest in any future study. Taking into account the findings of recent research on psycho-neural pathways that show promise in explaining socio-economic patterning of health outcomes,[58, 59] it may be possible to determine more proximal physiological outcomes, such as cortisol levels, which could be assessed non-invasively and might offer a more sensitive signal. However, such data are unlikely to be available routinely.

## Supporting information

S1 Tables Supplementary tables A, B and C. (DOCX)

S1 Text TIDieR checklist. (DOCX)

S2 Text Qualitative interview topic guide. (DOCX)
