# Supplementary material for: Does domiciliary welfare rights advice improve health-related quality of life in independent-living, socio-economically disadvantaged people aged ≥60 years? Randomised controlled trial, economic and process evaluations in the North East of England
Source: PLoS One. 2019 Jan 10;14(1):e0209560. doi: 10.1371/journal.pone.0209560 (PMC6328099; doi:10.1371/journal.pone.0209560)
Supplement: S1 Tables — (DOCX) [file pone.0209560.s001.docx]

**Supplementary Table A: Detailed intervention description.[**[**22**](#_ENREF_22)**]**

| **Aspect of intervention** | **Detailed description** |
| --- | --- |
| Type of Intervention | Domiciliary welfare rights advice for people aged 60 years or over |
| Rationale and underpinning theory | There is a strong positive and progressive relationship between access to financial and material resources and health outcomes.[[2](#_ENREF_2)] Older people, especially those in poor health, may need additional income or support, such as payments for social care, transport, domestic help and aids and adaptations to their home to maintain their health and independence.[[4](#_ENREF_4)] Such social welfare benefits are hypothesised to improve health-related quality of life, mediated by reduced stress, the adoption of more advantageous social arrangements and healthier behaviours. These changes in turn are hypothesised to enable greater choice and control over life circumstances, leading to healthier choices and greater independence.[[13](#_ENREF_13)] Historically in the UK, there has been substantial under-claiming of such financial and non-financial social welfare benefits among those with low incomes and poor health.[[10](#_ENREF_10),[11](#_ENREF_11)] Domiciliary welfare rights advice services aim to support users to make successful benefit claims, hence increasing their income and access to material resources. |
| Details of the intervention procedures and materials | Welfare rights advice consultations and active assistance with benefit claims were offered and delivered in participants own homes, tailored to individual needs by a trained welfare rights advisor employed by a local government department or a local voluntary organisation in North East England.  Participants were given an appointment in their own home with a welfare rights advisor within 2 weeks of randomisation, during which participants undergo a full benefit entitlement assessment involving: assessment of financial, material and welfare status; assessment of previous benefit entitlement and claims; discussion of current entitlement and options for action, including new claims (financial and non-financial). Active assistance with benefit claims and other welfare issues are given. Complex claims or those referred for further assessment or tribunal are managed in the usual way by welfare rights advisors. Participants are followed up intermittently by welfare rights advisors until they no longer require assistance (cases are usually closed once all claims and appeals have been resolved satisfactorily). |
| Intervention provider and training | Welfare rights advisors were recruited from local government departments or a local voluntary organisation in North East England. Advisors remained employed by their department and were seconded to undertake intervention delivery for the duration of the trial.  Training for participating welfare rights advisors was provided before commencement of the trial by an experienced welfare rights advisor from a local government department in the North East of England who had participated in the pilot RCT. The training was delivered in a half-day workshop and comprised information on the challenges of under claiming among older people, the full range of financial and non-financial benefits entitlements available to people aged 60 years or older in England, the mechanisms for claiming and the principles and practice of active assistance with claiming. |
| Mode of delivery | Welfare rights advice was delivered in participants own homes at a mutually convenient time over one or more sessions as necessary, depending on the complexity of the participant’s circumstances and health status. Follow-up meetings were arranged as required to ensure completion of claims on a case by case basis. Some follow-up work was conducted by telephone where this was feasible, according to usual practice of welfare rights advisors. |
| Location of delivery | The trial took place in eight of the 12 local government districts in socioeconomically disadvantaged areas in the North East of England. The intervention was delivered in participants own homes, for the most part in face-to-face meetings between welfare rights advisor and client, with some follow-up contacts by telephone where appropriate. |
| Intervention schedule and timing | We aimed to give participants an initial appointment with their allocated welfare rights advisor within 2 weeks of randomisation. Length of visit was tailored to individual needs and could last from 1-3 hours. Further visits or follow-up phone calls were arranged as required to complete claims on behalf of clients over the following months. It was anticipated that approximately 50 per cent of claims would be resolved within 3 months, but some would take up to 12 months. |
| Tailoring | The intervention was tailored to individual needs by the welfare rights advisor, according to usual practice. In practice this meant identifying the welfare benefits to which a client was entitled by individual assessment and delivering advice and active assistance with claims, tailored to those entitlements and the expressed wishes of the client. |
| Modifications | Due to cuts to local government funding during the study, it was not possible for delivery of the intervention to every participant by a welfare rights advisor from their nearest local government department. In these circumstances, we ensured that a qualified and trained welfare rights advisor was allocated to every client from an alternative, nearby local government department. |
| Assessment of intervention adherence and fidelity | Intervention procedure checklists were given to all welfare rights advisors to ensure consistent delivery. We assessed intervention delivery and adherence by collecting data on every case, including timing and completion of welfare rights advice case work at each stage of the process.  We assess fidelity by audio-recording a sample of welfare rights advice sessions with participants (target: one per advisor (n=19)). These recordings were listened to by an experienced welfare rights advisor not involved in the study, who assessed fidelity according to a protocol. |
| Reported intervention adherence and fidelity | Number of intervention arm participants seen as intended within 2 weeks by their allocated welfare rights advisor was 5 (1.5%) and within 4 weeks 37 (11%).  Median number of days from study entry to first welfare rights advisor visit was 58 days (IQR: 40-89), range 0-403 days.  Length of time taken for welfare rights advisors to see participants for their initial assessment increased as recruitment progressed. The median time from recruitment to welfare rights advisor case being closed was 83 days (IQR and range: 51-140.5 and 14-705 days, respectively).  Of 381 intervention arm participants, 335 (88%) received the intervention as intended. For those not receiving the intervention (n=46, 12%), the most commonly cited reason was that the participant declined the WRA consultation (n=23); this represented 6% of those eligible and 50% of those not receiving the intervention. In addition, some participants withdrew from the study before intervention delivery (n=16), while some could not be contacted in order to arrange the advice appointment (n=7).  Seven recordings of welfare rights advisor initial assessments with participants in the intervention group were made available for fidelity assessment. All consultations were carried out systematically, were consistent with the protocol for intervention delivery and included appropriate assessment of financial and health status, and all relevant applications for eligible means and non-means tested awards and benefits. |

**Supplementary Table B: Secondary outcome measures**

| **Outcome domain** | **Measurement instrument** | **Description** |
| --- | --- | --- |
| Mental health status | Patient Health Questionnaire-9 (PHQ-9) depression scale[^1-3^](#_ENREF_1) | PHQ-9 examines nine mental health problems. The depression scale range is 0–27, with lower values indicating fewer depressive symptoms |
| Perceived financial well-being | Affordability Index[^4^](#_ENREF_4) | The 13-item index has a possible range of 4–20, with lower scores indicating fewer financial problems |
| Standard of living index | A scale assessing ownership of 24 household items, based on questions used in the British General Household Survey[^4^](#_ENREF_4) | Possible range is 0–24, with a higher score indicates a higher standard of living. |
| Social support and participation | Measured by questions examining social interaction and strength of confiding relationships[^5^](#_ENREF_5) | The social interaction score has a possible range of 0–27, with higher scores indicating a higher level of social engagement and support. An isolation indicator was created from one item on this scale, categorising whether or not participants reported that they did not see friends and relatives as often as they wished. |
| General health status | Measured by the EuroQol-5 Dimensions-3 Levels (EQ-5D-3L) instrument[^6^](#_ENREF_6)^,^[^7^](#_ENREF_7) | The EQ-5D-3L is a five-item scale with three levels of response. The EQ-5D-3L is a self-completion questionnaire comprising five dimensions of quality of life: mobility, self-care, usual activities, pain/discomfort and anxiety/depression. The tool has been extensively used in studies and full details are reported on the website of the EuroQoL Group ([www.euroqol.org/](http://www.euroqol.org/)). |
| Health-related behaviours | Assessed by self-report, to measure change in key indicator behaviours, such as smoking, alcohol consumption,[^8^](#_ENREF_8) diet (consumption of key food groups)[^9^](#_ENREF_9) and the Physical Activity Scale for the Elderly (PASE)[^10^](#_ENREF_10) | Smoking was recorded …  Alcohol consumption was recorded…  The diet score had a possible range of 15–75, with higher scores indicating a healthier diet in terms of salt, fat and sugar consumption.  The physical activity score had a possible range of 0–400+, with higher scores indicating higher levels of activity. |
| Mortality | Certified cause of death | Assessed by identifying deaths at 12 months and 24 months from GP records. |
| Financial status | Assessment tool developed and used in our pilot RCT[^5^](#_ENREF_5) | Tool assessed all sources of household income, including benefits, major outgoings (rent/mortgage, fuel bills, etc.), debts and capital assets (i.e. home and savings). As well as these data, at follow-up detailed data were collected (by WRAs) on new benefits received since baseline, including one-off (lump sum) payments and regular, weekly or monthly income. |
| Independence | Measured by assessing living arrangements and carer status | Categories: living independently or with carer support, in own home, with relations, in a care home or hospital. We also assessed (by self-report) the number of hours of home care received per week. |

**References for Supplementary Table A2**

**1.** Cameron I, Crawford JR, Lawton K, Reid IC. Psychometric comparison of PHQ-9 and HADS for measuring depression severity in primary care. *British Journal of General Practice.* 2008;58:32-36.

**2.** Kroenke K, Spitzer RL. The PHQ-9: A New Depression Diagnostic and Severity Measure. *Psychiatric Annals.* 2002;32(9):1-7.

**3.** Lowe B, Kroenke K, Herzog W, Grafe K. Measuring depression outcome with a brief self-report instrument: sensitivity to change of the Patient Health Questionnaire (PHQ-9). *Journal of Affective Disorders.* 2004;81:61-66.

**4.** Ford G, Ecob R, Hunt K, Macintyre S, West P. Patterns of class inequality in health through the lifespan: class gradients at 15, 35 and 55 years in the west of Scotland. *Soc Sci Med.* 1994;39(8):1037-1050.

**5.** Mackintosh J, White M, Howel D, et al. Randomised controlled trial of welfare rights advice accessed via primary health care: pilot study. *BMC Public Health.* 2006;6(162).

**6.** Szende A, Oppe M, Devlin N, eds. *EQ-5D Value Sets: Inventory, Comparative Review and User Guide.* Berlin: Springer; 2007. EuroQol Group Monographs; No. 2.

**7.** The EuroQol Group. EuroQol-a new facility for the measurement of health-related quality of life. *Health Policy* 1990;16(3):199-208.

**8.** Sobell L, Sobell M. *Alcohol Timeline Followback Users’ Manual.* Toronto, Canada1995.

**9.** Roe L, Strong C, Whiteside C. Dietary intervention in primary care: validity of the DINE method for dietary assessment. *Fam Pract.* 1994;11:375-381.

**10.** Washburn RA, Smith KW, Jette AM, Janney CA. The Physical Activity Scale for the Elderly (PASE): development and evaluation. *J Clin Epidemiol.* 1993;46:153-162.

**Supplementary Table C:** **Comparison of characteristics of participants who did or not receive additional benefits at baseline and 24 months (Intervention arm only)**

|  |  | **Benefits awarded and received  (n=84)** | | | | **No benefits awarded ^a^  (n=267)** | | | |
| --- | --- | --- | --- | --- | --- | --- | --- | --- | --- |
| **Categorical variables** | **Categories** | **Number (%)** | | | | **Number (%)** | | | |
| **Sex** | **Male** | 31 (41) | | | | 121 (47) | | | |
| **Education level** | **Primary**  **Secondary**  **Tertiary** | 1 (1)  67 (89)  7 (10) | | | | 3 (1)  209 (81)  47 (18) | | | |
| **Marital status** | **Living alone** | 41 (55) | | | | 111 (43) | | | |
| **Accommodation** | **Not paying** | 42 (59) | | | | 154 (61) | | | |
| **Continuous variables** |  | **n** | **Mean** | **SD** | **Range** | **n** | **Mean** | **SD** | **Range** |
| **Age in years** |  | 84 | 71.4 | 6.8 | 60-88 | 266 | 69.7 | 7.2 | 60-92 |
| **Townsend ADL score at 24 months** |  | 60 | 8.5 | 4.1 | 0-16 | 195 | 12.2 | 4.1 | 0-16 |
| **Life events score at 24 months** |  | 66 | 4.0 | 3.6 | 0-15 | 209 | 4.7 | 4.1 | 0-18 |
| **IMD score^b^** |  | 83 | 29.5 | 16.3 | 6.2-71.7 | 266 | 29.0 | 16.3 | 3.4-74.5 |

^a^ No benefits because none awarded or eligible for benefits but refused them

^b^ Index of Multiple deprivation based on LSOA: Higher scores indicate greater deprivation
