# Supplementary material for: Does domiciliary welfare rights advice improve health-related quality of life in independent-living, socio-economically disadvantaged people aged ≥60 years? Randomised controlled trial, economic and process evaluations in the North East of England
Source: PLoS One. 2019 Jan 10;14(1):e0209560. doi: 10.1371/journal.pone.0209560 (PMC6328099; doi:10.1371/journal.pone.0209560)
Supplement: S2 Text — (DOCX) [file pone.0209560.s003.docx]

**
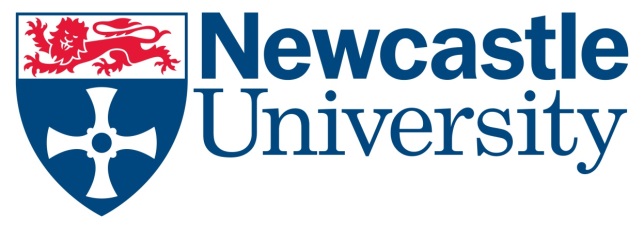
**

***Trial of welfare advice for older general practice patients (the Do-Well study*)**

**Qualitative study**

**Topic guide – intervention group +extra resources**

**This study is about helping people claim the help that they are entitled to. We are interested to find out your views on getting welfare rights advice as part of the GP service and any effects it might have had on people’s lives. I’ll be asking you a number of questions about this.**

- **Everything that you say will be confidential, and no names will appear in any reports about the study.**
- **With permission – audio record**
- **If there are any questions you don’t want to answer, then you don’t have to.**
- **If you would like to stop the interview at any point, then just say.**

**Background – help received**

Can you tell me when you had your interview with the welfare rights advisor ?

Can you tell me about any extra benefits or help you have received as a result of this advice?

What do you think about the help that you got from the welfare advice officer?

*Probe:*

*useful*

*not useful*

**Prior dealings/knowledge of welfare rights advice**

Before you saw the welfare rights advisor this time, had you received any welfare advice before?

*Probe:*

*Did you go to an advice centre/CAB?*

*What advice did you receive?*

*What was it like?*

*How did it compare to the service offered by Rosemary?*

How did you feel about getting a letter about welfare advice from your general practice?

Before you got your letter, did you feel confident that you knew what help you were entitled to?

**Entitlement – knowledge**

Do you think it’s easy to know what help you’re entitled to?

Were you surprised to get what you got?

Did you have any concerns about claiming?

*Probe:*

*Worried because not entitled*

*Worried about level of intrusion*

*Worried about confidentiality*

If you hadn’t received the extra benefit/help how would you have felt?

*Probe:*

*Blamed anyone?*

*Hopes raised and dashed?*

*Tried again?*

Did you talk to your GP about this claim?

Did you talk to anyone else before going ahead?

Did you think that making this claim would affect your relationship with your GP?

**Impact of additional resources**

How has getting the extra benefit/service affected you?

Have things changed in any way?

Is there anything you can do now that you couldn’t do before?

*Probe:*

*Worry/anxiety*

*Depression/mood*

*Smoking*

*Alcohol*

*Diet*

*Activity levels*

*Social benefits*

(If financial)

Can you say how you use the extra money?

**We are interested in finding out what people think affects their health**

What things do you think most affect your health?

What things do you think would make you more healthy?

What things do you think make you feel less healthy?

What makes you feel healthy ?

*Probe:*

*Work*

*Relationships*

*Specific illness*

*Housing*

*Condition of the area you live in*

Do you think that your health has been affected in any way since you received welfare advice?

Do you think that money has anything to do with health?

*Probe:*

*Do you think that having more money would make you healthier?*

*In what way would it/wouldn’t it?*

**Thank you very much for your help and time, do you have any further questions?**
